# Do COVID-19 cases follow a similar transition path? Evidence from Indian states

**DOI:** 10.1016/j.mex.2020.101196

**Published:** 2020-12-23

**Authors:** Vaseem Akram, Badri Narayan Rath, Pradipta Kumar Sahoo

**Affiliations:** a Economics and Business Environment Area, Indian Institute of Management Jammu, Jammu and Kashmir 180016, India; b Department of Liberal Arts, Indian Institute of Technology Hyderabad. Kandi, Sangareddy, Telangana 502285, India

**Keywords:** COVID-19, Club convergence, Indian states

## Abstract

This paper assesses the convergence of COVID-19 cases by obtaining transition paths of Indian states covering the period from August 01 to October 31, 2020. The results based on Phillips-Sul test show evidence of different transition paths. These findings are useful from the policy perspective, particularly to see whether existing efforts made for stopping the spread of COVID-19 by states/central governments are effective.

• Convergence of COVID-19 cases across Indian states is investigated.
• The Phillips and Sul test is applied.
• Findings are in favour of different transition paths.

## Graphical abstract

**Figure:**
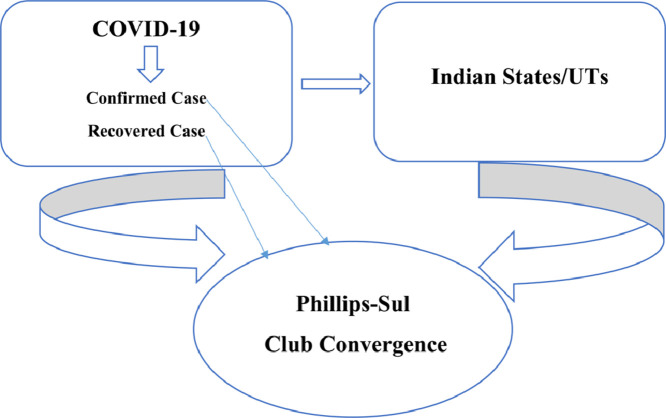

Specifications table

**Table:** 

Subject Area:	Economics and Finance
More specific subject area:	COVID-19 club convergence
Method name:	Phillips and Sul club convergence test
Name and reference of original method:	The original method is reported in [29] P.C.B. Phillips, D. Sul, Transition modeling and econometric convergence tests, Econometrica. 75(6) (2007) 1771–1855.
Resource availability:	K. Du. Econometric convergence test and club clustering using Stata. Stata J. 17(2017) 882–900. https://doi.org/10.1177/1536867X1801700407.

## Introduction

The novel coronavirus disease (COVID-19) has been spreading rapidly across the world [1]. India ranked as the second in COVID-19 cases with around 8.41 million and 123,611 cases of death as of 04 November 2020 (Ministry of Health and Family Welfare, Government of India). To curb the outbreak, various steps have been undertaken by both central and state governments. Despite several efforts made by governments, an unevenness in confirmed and recovered cases has been noticed. As a result, some states are severely affected by COVID-19, and some are less affected. For instance, Maharashtra, Delhi, Karnataka, and Tamil Nadu are relatively more affected states. Contrastingly, it is observed that states/ Union Territories (UTs) like Arunachal Pradesh, Mizoram, and Sikkim are the least affected.

There is widespread of literature, which focus on the impact of COVID-19 on key macroeconomic indicators. For instance, a set of studies investigates the COVID-19 impact on crude oil prices, corporate performance, stock markets, energy, etc. [2], [3], [4], [5], [6], [7], [8], [9], [10], [11], [12], [13], [14], [15], [16], [17], [18], [19], [20], [21], [22], [23], [24]. Few studies also explore the COVID-19 impact on firms/industry/sectors [25], [26], [27]. Moreover, [28] examine the convergence of COVID-19 cases and found that control of the spread among the countries is weak, but a detailed study on COVID-19 convergence at the sub-national level is scanty.

Our study fills this research gap by complementing the above studies into three folds. First, none of the above studies discusses the growth and transition path of COVID-19 cases across Indian states. The investigation of COVID-19 convergence is crucial in the context of India from a readiness perspective as India is unlocking phase-wise to bring its economy back to track. Second, this study complements the [28] study by investigating the convergence based on clustering algorithms. To do so, we use Phillips and Sul (hereafter, PS) [29] techniques that make the clubs based on the COVID-19 cases of states’ speed of convergence. Third, this study uses confirmed and recovered cases as a measure of COVID-19 and compute the speed of convergence for both indicators.

The study is systemized as follows. Section 2 illustrates the methodology and data. Section 3 offers empirical results and the final section 4 concludes.

## Method details

### Methodology

To identify the transition paths of COVID-19, this study implements a novel approach propounded by Phillips and Sul [29]. This test is based on the clustering algorithm that clusters the club of countries or states based on transition paths. Moreover, this method considers the nonlinear time-varying factor unlike the Solow-Swan growth model [30,31]. The most important feature of this test is that it does not count the “cointegration or unit root properties” and it considers the transitional dynamics in addition to long-run behaviour of the variable. In other words, it amends the bias that ascends from both “stationary and non‐stationary or a mix of both series in the panel due to misspecification”. All conventional unit root tests and simple regression may not account the heterogeneity of cross-sections as a result, they may provide misleading results [32]. Thus, PS include both “common and cross-section” specific behaviour and find multiple steady-states (club convergence) unlike “beta and sigma convergence”, which is built on a steady-state.

PS test is quite familiar in the recent empirical studies of convergence. This study uses the PS test to examine convergence and transition paths of COVID-19 cases by using subnational data of India. We begin by writing the single factor model as follows:

(1) $$\text{COVID}-19_{\text{it}}=\delta_{i}\mu_{t}+u_{\text{it}}$$

Where $\text{COVID}-19_{\text{it}}$ considered as confirmed and recovered cases of COVID-19 in the case of Indian states (i) at time (t). In Eq. (1), $\;\delta_{i}$ refers to the “idiosyncratic distance” between the common factor $\mu_{t}\;$and systematic part of $\text{COVID}-19_{\text{it}}$. $\mu_{t}$ stands for common behaviour of $\text{COVID}-19_{\text{it}\;}$ and $u_{\text{it}}$ represents error term. Further, Eq. (1) is decomposed into $A_{\text{it}}$ which refers to the “systematic” components and $B_{\text{it}}$ refers to “transitory” components.

(2) $$\text{COVID}-19_{\text{it}}=A_{\text{it}}+B_{\text{it}}$$

Eq. (2) can be further expanded as:

(3) $$\text{COVID}-19_{\text{it}}=\left(\frac{A_{\text{it}}+B_{\text{it}}}{u_{t}}\right)u_{t}=\delta_{\text{it}}u_{t},$$

Where $\delta_{\text{it}}\;$indicates “idiosyncratic element” and $u_{t}$ is a single “common steady-state trend function” which captures some stochastic and deterministic trend behaviour. Furthermore, coefficients of $\delta_{\text{it}\;}$show the common factor share of ($\mu_{t})$ each cross-section in a group of cross-sections. The convergence approach based on PS follows a dynamic-process and $\delta_{\text{it}}\;$advises “transition paths”. The model cannot be fitted without imposing few restrictions on $\delta_{\text{it}}$ and $u_{t}$. Thus, PS removes the common factors by following the below expression.

(4) $$h_{\text{it}}=\frac{\text{COVID}-19_{\text{it}}}{\frac{1}{N}\sum_{i=1}^{N}\text{COVID}-19_{\text{it}}}=\;\frac{\delta_{\text{it}}}{\frac{1}{N}\sum_{i=1}^{N}\delta_{\text{it}}}$$

Where, $h_{\text{it}}$ refers to relative transition coefficient at time ‘t’ and $h_{\text{it}}$ varies between the cross-section in short-run but achieve the convergence in long-run when $h_{\text{it}}\to 1$ for all i, when t→∞. This arises in the long run when cross-section variance is zero ($h_{\text{it}}\to 0)$. Thus, PS test suggests the following assumption for convergence (club) for the “semiparametric form of time-varying coefficients $\delta_{\text{it}}$”:

(5) $$\delta_{\text{it}\;}=\delta_{i\;}+\sigma_{\text{it}}\xi_{\text{it}}$$

In Eq. (5),$\;\sigma_{\text{it}}$ is defined as the $\frac{\sigma_{i}}{L\left(t\right)t^{\alpha }}.\;$Where $\sigma_{i}>0,$ and t≥1. $\xi_{\text{it}}$ show the “weakly dependent” over-time (t). Moreover, it is independent and identically distributed (0, 1) over-cross-sections (i). L(t) function varies slowly. It converges and diverges to infinity [L(T)→∞ as t→∞].

PS test follows a null hypothesis of convergence $H_{0}:\delta_{i\;}=\delta ,\;\text{with}\;a\geq 0$, and $H_{1}:\delta_{i\;}\neq \delta ,\;\text{with}\;a<0$. Where the final regression is implemented by [29] is written as follows:

(6) $$\text{log}\left(\frac{\delta_{h1}^{2}}{\delta_{\text{ht}}^{2}}\right)-2\;\text{log}\left[\text{log}\left(t\right)\right]=\alpha +\beta \text{log}\left(t\right)+u_{t}$$

In Eq. (6), $\frac{\delta_{h1}^{2}}{\delta_{\text{ht}}^{2}}$ refers to the cross-section's variance ratio (or states’ variance ratio in our case). Where tis defined as the [rT],[rT]+1,…,T with r>0. Based on the Monte Carlo experiments, the PS test suggests that r∈(0.2,0.3).Specifically, it is recommended to set r=0.3 for the small (*T* ≤ 50) sample and set r=0.2 for the large *T* ≥ 100) sample. The t-test statistic is based on “standard normal distribution” asymptotically that is created utilizing the estimated β.

### Data

The data on confirmed and recovered cases have been collected from CEIC India premium database starting from August 01, 2020, to October 31, 2020. The choice of selecting the beginning period as August 01, 2020, is mostly based on the availability of reasonable COVID-19 positive cases and recovered cases data across the states/UTs.

### Empirical results

The existence of heterogeneity in COVID-19 cases across Indian states/UTs helped us to employ the PS test and the results of confirmed cases are reported in Panel A in Table 1. The results indicate that states/UTs in the aggregate panel are diverging in terms of confirmed cases as log(t) value (−138. 38) is less than the critical value (−1.65). This implies that states are not following same transition paths rather, there exist distinct transition paths. However, the presence of convergence is noticed at the club level. Club 1 includes mostly those states/UTs, which are having a large number of confirmed cases. Club 2 encompasses those states/UTs which are having relatively low confirmed cases and Club 3 represents the least states/UTs in terms of confirmed cases. We also identified one group (that includes Nagaland and Maharashtra), which is neither converging nor diverging. Further, we also examine the convergence for COVID-19 recovered cases and results are reported in Panel B in Table 1. The results show the existence of two clubs. Club 1 includes the highest recovery states and club 2 encompasses those states that have the least recovery rate but follow same transition path.

**Table 1:** Results of convergence of COVID-19 confirmed cases.

Clubs	States/UTs	$t_{\hat{\beta }}$	log(t)	Decision	SPC
**Panel A: Confirmed Cases**					
Full samples	All states	−0.29	−138. 38	Divergence	—
Club 1	Kerala, Madhya Pradesh, Uttar Pradesh, Andhra Pradesh, Chhattisgarh, Delhi, Karnataka, Odisha, Tamil Nadu, Uttarakhand, West Bengal	0.222**	19.228	Convergence	0.111
Club 2	Assam, Gujarat, Tripura, Arunachal Pradesh, Bihar, Chandigarh, Goa, Haryana, Himachal Pradesh, Jammu and Kashmir, Jharkhand, Manipur, Meghalaya, Puducherry, Punjab, Rajasthan, Telangana	0.041**	17.788	Convergence	0.021
Club 3	Dadra and Nagar Haveli and Daman and Diu, Andaman and Nicobar Islands, Ladakh, Mizoram, Sikkim,	0.390**	31.319	Convergence	0.195
Group	Nagaland, Maharashtra	−0.340	−59.320	Not convergence	—
**Panel: B Recovered cases**					
Full samples	All states	−0.19	−187.14	Divergence	—
Club 1	Assam, Gujarat, Kerala, Madhya Pradesh, Tripura, Uttar Pradesh, Andhra Pradesh, Arunachal Pradesh, Bihar, Chandigarh, Chhattisgarh, Delhi, Goa, Haryana, Himachal Pradesh, Jammu and Kashmir, Jharkhand, Karnataka, Meghalaya, Odisha, Puducherry, Punjab, Rajasthan, Tamil Nadu, Telangana, Uttarakhand, West Bengal	0.093**	24.229	Club convergence	0.047
Club 2	Dadra and Nagar Haveli and Daman and Diu, Nagaland, Andaman and Nicobar Islands, Ladakh, Mizoram, Sikkim	0.910**	18.828	Club convergence	0.455
Group	Maharashtra, Manipur	−0.270	−81.849	Not convergence	—

*Notes:* The critical value is −1.65 at 5% level of significance level. **Indicates non-rejection of the null of convergence. The results show evidence of club convergence. SPC indicates the speed of convergence.

**Fig. 1:**
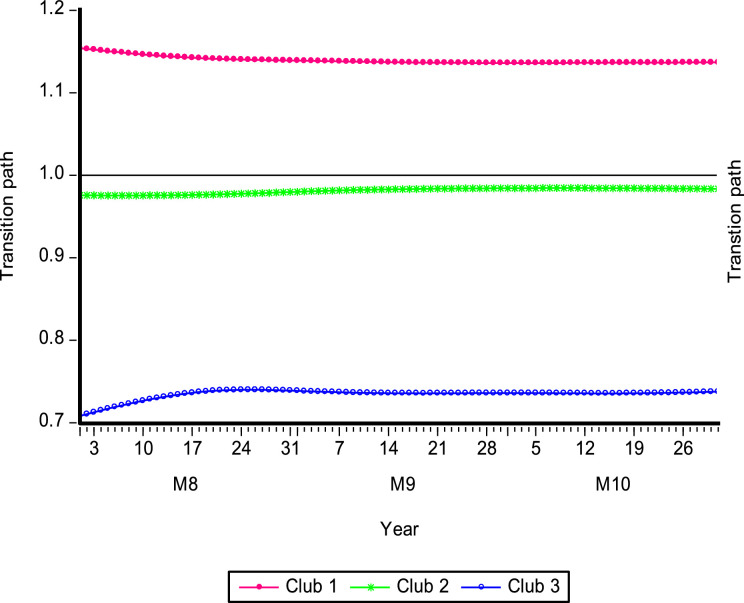
Transition paths of confirmed cases. In this figure, we plot the transition paths of the confirmed cases across the clubs using equation (4). It is noted that states which lie in club 1, have the highest confirmed cases as compared to club 2 and 3. Clubs 2 and 3 are below the average. Club 2 transition path is below the average of all states/UTs (straight line) but closer to average. Whereas, club 3 states’ transition path is extremely below the average of all states/UTs. This implies that states which fall in club 3 have the lowest confirmed cases. Overall, the graph shows that each club follows a distinctive transition path. Source: Authors calculation based on PS test.

**Fig. 2:**
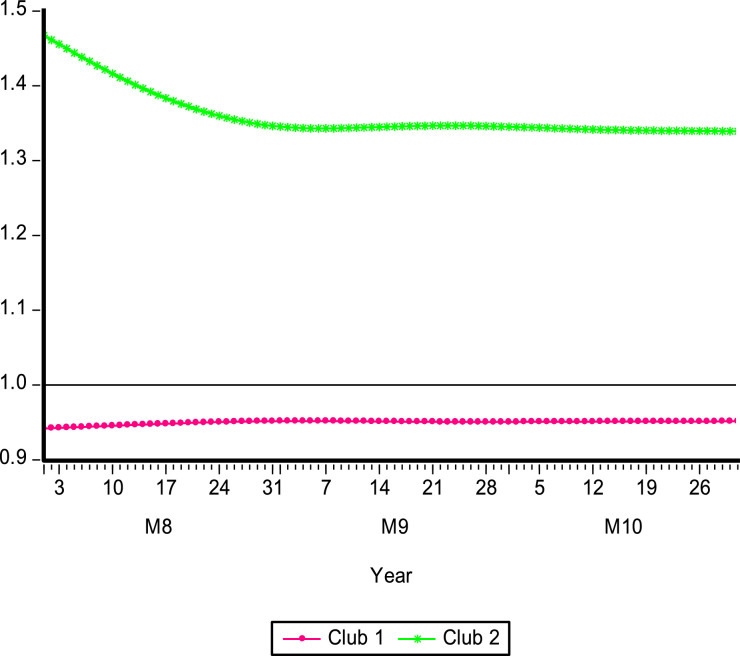
Transition paths of recovered cases. In this figure, we plot the transition paths of the recovered cases across the clubs using equation (4). We observed that states which lie in club 1 are having highest recovered cases as compared to club 2. However, both club 1 and club 2 follow distinct transitions path. Source: Authors calculation based on PS test.

Then to visualize the transitions paths of confirmed cases, we compute the relative transition paths by using Eq. (4). In Panel-A, we find three different transition paths for Clubs 1, 2, and 3 and each clubs' transition path is unique. In Fig. 1, we notice that the transition path of club 1 is above the average of all the states/UTs (marked with a straight line at value 1). Club 2′s transition path is below the average of all states/UTs but it is almost closer to average. Whereas, club 3 states’ transition path is extremely below the average of all states/UTs. We plot the transition paths for recovered cases in Fig. 2 and our results show that states are following distinct transition paths.

We then compute the speed of convergence (SPC) for both confirmed and recovered cases. SPC is labelled in column fifth in Table 1. The highest SPC for the confirmed case and recovered case are noticed in Club 3 and Club 2 respectively. To sum up, although several measures have been taken by the governments to stop the spread of COVID-19, results clearly indicate that states are following different transition paths with a varying speed of convergence.

## Conclusions

This present study investigates the COVID-19 convergence in case of 28 Indian states and 7 Union Territories. The results based on Phillips and Sul test show evidence in favour of divergence for both confirmed and recovered cases for the aggregate panel. This implies that states and UTs are following different transitions paths. Further, evidence of convergence of COVID-19 positive cases and confirmed cases are noticed at the club level. These findings are useful from the policy perspective, particularly to see whether existing efforts made for stopping the spread of COVID-19 by states/central governments are effective.

## Declaration of Competing Interest

The Authors confirm that there are no conflicts of interest.
